# Suppressive effect of azithromycin on *Plasmodium berghei *mosquito stage development and apicoplast replication

**DOI:** 10.1186/1475-2875-9-73

**Published:** 2010-03-10

**Authors:** Shoichi Shimizu, Yoshio Osada, Tamotsu Kanazawa, Yoshiya Tanaka, Meiji Arai

**Affiliations:** 1 Department of Immunology and Parasitology, University of Occupational and Environmental Health, Japan, 1-1 Iseigaoka, Yahatanishi-ku, Kitakyushu 807-8555, Japan; 2 First Department of Internal Medicine, University of Occupational and Environmental Health, Japan, 1-1 Iseigaoka, Yahatanishi-ku, Kitakyushu 807-8555, Japan; 3 Department of International Medical Zoology, Faculty of Medicine, Kagawa University, 1750-1 Ikenobe, Miki-cho, Kita-gun, Kagawa 761-0793, Japan

## Abstract

**Background:**

Azithromycin (AZM) is a macrolide antibiotic that displays an excellent safety profile even in children and pregnant women and has been shown to have anti-malarial activity against blood stage *Plasmodium falciparum*. This study evaluated the transmission-blocking effect of AZM using a rodent malaria model.

**Methods:**

AZM-treated mice infected with *Plasmodium berghei *were exposed to *Anopheles stephensi *mosquitoes, followed by the observation of parasite development at different phases in the mosquito, i.e., ookinetes in the midgut, oocysts on the midgut, and sporozoites in the midgut and salivary glands. Furthermore, to evaluate the effect on organelle replication of each stage, quantitative real-time PCR analysis was performed.

**Results:**

The inhibitory effect of AZM was noticeable in both gametocyte-ookinete transformation in the midgut and sporozoite production in the oocyst, while the latter was most remarkable among all the developmental phases examined. Real-time PCR analysis revealed that AZM suppressed apicoplast replication at the period of sporozoite production in oocysts.

**Conclusions:**

AZM inhibits parasite development in the mosquito stage, probably through the same mechanism as in the liver and blood stages. Such a multi-targeting anti-malarial, along with its safety, would be ideal for mass drug administration in malaria control programmes.

## Background

Malaria, caused by protozoan parasites of the genus *Plasmodium *and transmitted by mosquitoes of the genus *Anopheles*, remains one of the world's most important health problems, causing nearly a million deaths per year [1]. Because of the rapid emergence and spread of drug-resistant *Plasmodium falciparum*, the development of alternative control tools is needed urgently [2]. A possible strategy is to block malarial transmission from gametocyte carriers to the vector mosquitoes using a transmission-blocking vaccine [2,3] or a drug that interrupts parasite development in the mosquito vector [4]. The strategy of blocking malarial transmission has been claimed to limit the spread of malaria and to reduce the spread of drug-resistant parasites [5-8].

Considering that very high coverage is essential for a significant impact on malaria transmission, mass drug administration (MDA) for people including children and pregnant women in endemic area is required [6]. In MDA, safety is a paramount issue because the drug will be given to large numbers of non-infected individuals [7]. Thus, only drugs with an excellent safety profile should be considered for MDA. Primaquine, the generally available gametocytocidal drug, has been used previously in MDA [9,10], but its haemolytic effect in glucose-6-phosphate dehydrogenase deficient individuals has made this drug less acceptable for MDA [7,8]. To date, a limited number of drugs or compounds has been confirmed to possess transmission-blocking activity [5,11-13]. Most of them have not been considered for clinical applications due to their toxicity and/or cost of development [12]. Therefore, if licensed antibiotics are proven to have transmission-blocking activity, practical evaluation should be greatly accelerated and their impact should be fully exploited [14].

Azithromycin (AZM), a 15-membered azalide that has been broadly used for the treatment of bacterial infections [15], displays a good safety profile, including in children and pregnant women [2,16,17]. Importantly, AZM was shown to have inhibitory activity against *Plasmodium *spp. both *in vitro *and *in vivo *[15,18-20] and was effective for prophylaxis and treatment against human malaria in field conditions [21-23]. It has been proposed that AZM exerts a "delayed death" effect against blood stage *P*. *falciparum*, in which the progeny of AZM-treated parasites that inherited non-functional apicoplasts fail to develop, leading to a delayed, but potent anti-malarial effect [24]. Therefore, it was hypothesized that this delayed effect of AZM would also be exerted against parasite development in the mosquito, leading to transmission blockade.

The aim of this study was to evaluate the transmission-blocking activity of AZM using a rodent malaria model, and to investigate whether the apicoplast is the target of AZM in the mosquito stage.

## Methods

### Parasites and mosquitoes

*Plasmodium berghei *ANKA strain, clone 2.34 was maintained by cyclic passage in BALB/c mice (Japan SLC, Shizuoka, Japan) and SDA500 strain of *Anopheles stephensi*. For drug treatment and mosquito biting studies, mice infected with the second blood passage parasites after mosquito transmission were used. Cyclic colonies of *An. stephensi *were maintained at University of Occupational and Environmental Health, Japan. Mosquitoes were reared according to the MR4 Methods in *Anopheles *Research Manual [25].

### Administration of AZM to *P. berghei*-infected mice

All the animal experiments were performed under the control of the Ethics Committee of Animal Care and Experimentation in accordance with The Guiding Principles of Animal Care Experimentation, The University of Occupational and Environmental Health, Japan and the Japanese Law for Animal Welfare and Care (No. 221). Six-week-old female BALB/c mice (18.5-20.5 g) were infected with *P. berghei *by intraperitoneal injection of 5 × 10^6 ^parasitised erythrocytes per mouse. Parasitaemia and mature microgametocytaemia (density of male gametocytes), the latter of which is thought to be a limiting factor for the efficiency of fertilization in the vector mosquito due to the female-biased sex ratio [26,27], were monitored daily by microscopic observation of Giemsa-stained thin blood smears [14]. Four days post-infection, 6-10 infected mice were divided into two groups to match parasitaemia and microgametocytaemia. Mice of the experimental group were given azithromycin (AZM) (LKT Laboratories, St. Paul, MN, USA) suspended in 0.3% carboxymethylcellulose (CMC) (Nacalai Tesque, Kyoto, Japan) at a dose of 400 mg/kg orally and those of the control group were given 0.3% CMC only.

### Exflagellation assay

In order to evaluate the effect of AZM on exflagellation (microgametogenesis) activity, 3 μl of tail blood was taken from each infected mouse at 24 hours after drug administration (the same time as blood feeding). The blood sample was immediately mixed with 300 μl of exflagellation medium (RPMI 1640 containing 25 mM HEPES, 25 mM sodium bicarbonate, 20% foetal bovine serum, pH 8.0) and 20 μl of the suspension was loaded onto a Fuchs-Rosenthal haemocytometer (C-Chip DHC-F01, Digital Bio Technology, Seoul, Korea), then incubated at 19°C for 20 minutes. Exflagellation centres were numerated by microscopic observation at ×200 magnifications and expressed as a number of exflagellation events per 10^4 ^red blood cells (RBCs).

### Assessment of sporogonic development

At 24 hours post-drug administration, infected mice were exposed to 300-400 mosquitoes in each group that had emerged 6-8 days before, for 15 minutes at 19°C. Two- to three-hundred engorged mosquitoes in each group were collected and kept at 19°C. At 24 hours post blood feeding, 10-20 mosquitoes in each group were dissected for ookinete counting. The gut contents of each mosquito were mixed with fetal bovine serum on a glass slide. A thin film of the midgut contents was prepared and stained with Giemsa, then the ookinete count was made in a total of 300 observation fields with ×50 oil-immersion objective and ×10 ocular lens as described previously [28]. At 10 days post blood feeding, 10-20 mosquitoes in each group were dissected and the number of oocysts per midgut was determined by light microscopic observation. At 20 days post blood feeding, 30 mosquitoes in each group were dissected for sporozoite numeration. Ten sets of salivary glands and midguts were dissected out and pooled separately in 1.5 ml microtubes, then homogenized in 100 μl PBS. The suspension was loaded onto a haemocytometer, and the number of sporozoites was determined. Three replicates were performed for each experimental set. In another set of experiment, 20 mosquitoes from each group were individually dissected at 20 days post blood feeding, and the prevalence of salivary gland sporozoites was examined.

### Quantitative real-time PCR analysis to determine effect of AZM on apicoplast DNA replication

In order to evaluate the effect of AZM-treatment on apicoplast DNA replication, apicoplast DNA/nuclear DNA ratios were determined by quantitative real-time PCR. Blood stage genomic DNA of *P. berghei *was prepared from the mouse blood collected from 3-5 infected mice by cardiac puncture at 24 or 72 hours post-drug administration. For preparation of mosquito stage genomic DNA, midguts were dissected out and pooled from 20 infected mosquitoes in each group at 5, 10, and 15 days post blood feeding. Genomic DNA was isolated from the parasites by using Get pure DNA Kit-Cell, Tissue (Dojindo Molecular Technologies, Kumamoto, Japan) and Dr. GenTLE Precipitation Carrier (Takara Bio, Shiga, Japan) in accordance with the manufacturer's instructions. The nuclear and organelle-specific genes that we used for quantification of the nuclear DNA, apicoplast DNA, and mitochondrial DNA were *fabI*, *tufA*, and *cytb*, respectively [15,29]. Primers used for the amplification of each gene were designed and checked for specificity by using Primer-BLAST provided by National Center for Biotechnology Information [30]. The accession numbers of each gene and the designed primer sequences are listed in Additional file 1. Quantitative PCR was performed using Fast Real-Time SYBR Green master mix and a StepOnePlus sequence detection system (Applied Biosystems, Foster City, CA, USA) in accordance with the manufacturer's instructions. PCR was performed in duplicate for each sample. DNA samples obtained from blood stage parasites in the control group, which resulted in highest amount of amplified product for each gene, were serially diluted and used as the standard for absolute quantification. Organelle DNA replication was evaluated by comparison of PCR product quantities from organelle DNA with nuclear DNA [31,32]. To confirm specific amplification of desired products, DNA melting curve analysis was performed.

### Statistical analysis

Mann-Whitney U test was used to determine statistical differences in parasite number, parasitaemia and microgametocytaemia between the experimental and control groups. *P *values lower than 0.05 were considered significant. All tests were two-tailed. All statistical analyses were performed using Microsoft Excel 2007 (Microsoft Corporation, Tokyo, Japan).

## Results

### Determination of appropriate timing for blood feeding

It has already been reported that AZM exerts schizontocidal effects against *P. berghei *blood stage four days after drug administration [19]. Thus, to evaluate the effect of AZM against mosquito stage, the earlier timing seems to be appropriate for blood feeding. Therefore, at first, the parasitaemia and microgametocytaemia were checked at 24 hours post-drug administration. No significant difference between AZM-treated mice and control mice was observed in either parasitaemia (median value: 6.0% in AZM-treated mice vs 5.7% in control mice, *P *= 0.72, *n *= 20) or microgametocytaemia (0.031% vs 0.033%, *P *= 0.89, *n *= 20). Then, it was decided to treat mice with AZM 24 hours prior to blood feeding.

### Effect of AZM on exflagellation activity of *P. berghei *microgametocytes

**Figure 1:**
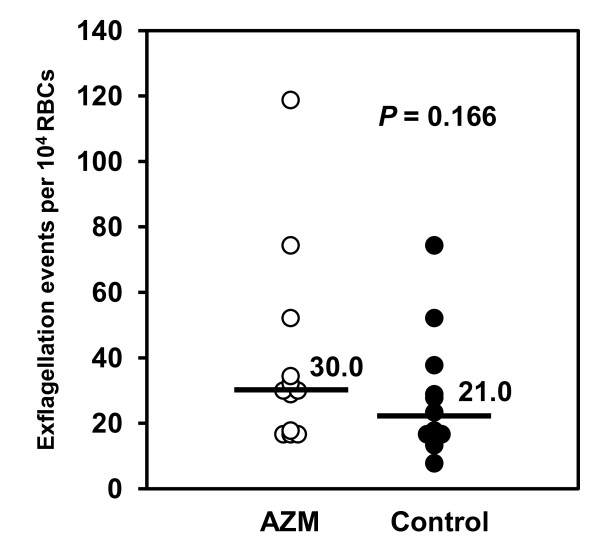
**Effect of AZM on exflagellation of *P. berghei *microgametocytes *in vitro***. The number of exflagellation events per 10^4 ^RBCs was counted *in vitro *24 hours after administration of AZM (White circle) or CMC (Black circle, as control). Horizontal lines and adjacent numbers represent the median number of exflagellation events in each group. *P *values were determined by Mann-Whitney U test. Combined results of three independent experiments are shown (*n *= 12).

To evaluate the effect of AZM on the function of microgametocyte, infected mouse blood was collected at the time of blood feeding (24 hours post-drug administration) and used to test for exflagellation activity. No significant difference of exflagellation events was observed between AZM-treated mice and control mice, suggesting that AZM did not affect the biological function of male gametocytes (Figure 1).

### Effect of AZM on mosquito stage development of *P. berghei*

**Figure 2:**
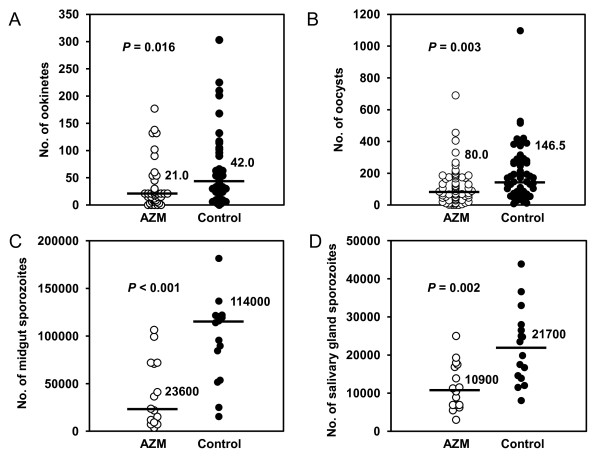
**Effect of AZM on *P. berghei *mosquito stage development**. Mosquitoes were allowed to feed on blood of *P. berghei*-infected mice that had been given AZM (White circle) or CMC (Black circle) at 24 hours before blood feeding. (A) Ookinete numbers in midguts dissected at 24 hours post blood feeding were determined by microscopy analysis. (B) Oocyst numbers on midguts dissected at 10 days post blood feeding were counted. (C, D) Sporozoite numbers in midgut oocysts (C) and salivary glands (D) were determined at 20 days post blood feeding. Horizontal lines and adjacent numbers represent the medians (*n *= 40 in (A); *n *= 60 in (B); *n *= 15 (calculated from 150 mosquitoes) in (C) and (D)). *P *values were determined by Mann-Whitney U test. Combined results of three to five independent experiments are shown.

The effect of AZM on parasite development in *An. stephensi *mosquitoes fed on blood of *P. berghei*-infected mice that had been given AZM 24 hours before blood feeding was assessed. Mosquitoes were dissected at 24 hours, 10, and 20 days post blood feeding to determine the levels of ookinetes, oocysts, and sporozoites both in the midgut and salivary glands, respectively. This experiment revealed that AZM treatment suppressed parasite development in all the four stages in the mosquito, i.e., reduction of ookinetes in the midgut (50% reduction as compared with the controls, *P *= 0.016, Figure 2A), oocysts on the midgut (45% reduction, *P *= 0.003, Figure 2B), sporozoites both in the midgut (79% reduction, *P *< 0.001, Figure 2C) and in the salivary glands (50% reduction, *P *= 0.002, Figure 2D). In another set of experiment, the prevalence of salivary gland sporozoites was 100% in both groups, indicating that AZM did not reduce the prevalence.

### Effect of AZM on replication of *P. berghei *apicoplast

**Figure 3:**
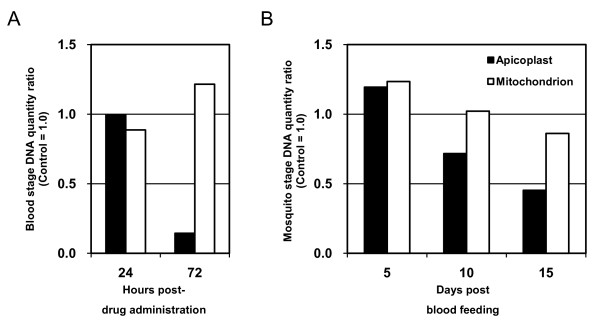
**Effect of AZM on replication of *P. berghei *apicoplasts**. Ratios of apicoplast DNA/nuclear DNA in AZM-treated parasites were calculated by quantitative real-time PCR, then standardized with the ratio from control parasites, giving ratios of (apicoplast/nuclear DNA ratio in AZM-treated parasites)/(apicoplast/nuclear DNA ratio in control parasites) (Black square). Similarly, the ratios of (mitochondrial/nuclear DNA ratio in AZM-treated parasites)/(mitochondrial/nuclear DNA ratio in control parasites) were calculated (White square). (A) Analysis of blood stage parasites at 24 hours post-drug administration (= the time of blood feeding) and 72 hours post-drug administration. (B) Analysis of mosquito stage parasites at 5, 10, and 15 days post blood feeding. DNA ratio of each control group is 1.0 on the vertical axis. A representative result of four independent experiments is shown.

In the case of blood stages, inhibition of apicoplast replication by chemicals has been usually evaluated by the ratio of apicoplast DNA to nuclear DNA [31,32]. In this study, to evaluate the effect of AZM on the replication of *P. berghei *apicoplast, the relative amounts of organellar DNA to nuclear DNA were estimated using real-time PCR. During the blood stage of parasite development, AZM treatment resulted in the suppression of apicoplast replication at 72 hours post-drug administration (1.0 at 24 hours post-drug administration, 0.14 at 72 hours post-drug administration, Figure 3A). This result agreed with previous reports about the effect of AZM [24], clindamycin [31], and 15-deoxyspergualin [32] on *P. falciparum in vitro*, all of which are believed to impair parasite growth by inhibiting apicoplast replication. In the mosquito stage, apicoplast replication in parasites derived from AZM-treated mice was not reduced at 5 days post blood feeding, i.e., 6 days post-drug administration, followed by successive suppressions at 10 days and 15 days post blood feeding (Figure 3B). In contrast, mitochondrial replication was not affected in both the blood and mosquito stages (Figure 3A, B), suggesting that the inhibitory effect of AZM was specific to the apicoplast.

## Discussion

It has been demonstrated that AZM inhibits apicoplast replication in blood stage *P. falciparum *[24] and liver stage *P. berghei *[33]. Indeed, combinations of AZM with conventional schizontocides were applied for human malaria cases in the field. They showed good results [22,34,35], whereas AZM monotherapy did not [34]. In this study, it was shown for the first time that AZM inhibits apicoplast replication of *P. berghei *also in the mosquito stage. These findings imply that AZM suppresses parasite growth at three different stages in the life cycle by attacking the same target, apicoplast replication. These data indicate that AZM has a further possibility of blocking malaria transmission.

In the present study, *P. berghei *development was apparently inhibited by AZM treatment at all the phases examined. It is notable that AZM treatment suppressed ookinete formation without affecting exflagellation activity, implying that AZM affected only macrogametocytes (female). This is compatible with the fact that the apicoplast is a maternally inherited organelle [36,37].

Considering the continuous development of parasites in mosquitoes, the reduction of oocysts (45%) was similar to the reduction of ookinetes (50%), suggesting that AZM does not have much effect on oocyst formation from ookinetes. On the other hand, the inhibitory effect of AZM on the mosquito stage was most remarkable in the production of sporozoites (79%). It has been reported in both blood and liver stage schizogony that replication and segregation of apicoplasts proceed during the middle to late stages of serial nuclear divisions and are completed before daughter merozoites are formed [33,38]. These findings allow it to be speculated that in the case of mosquito stage sporogony, apicoplast replication occurs during middle to late stage of nuclear divisions in oocysts, corresponding to around 8 to 15 days post blood feeding for oocyst maturation [39]. Therefore, inhibition of sporozoite production may result from the inhibition of apicoplast replication during the above-mentioned period in oocysts. Taken together, it seems that AZM inhibits mosquito stage development at two different steps; i.e., gametocyte-ookinete transformation in the midgut and sporozoite production in the oocyst. The latter is probably due to the inhibition of apicoplast replication. Interestingly, the drastic impact observed on midgut sporozoites was diminished at the salivary gland phase. A possible explanation for this discrepancy could be in part attributable to a linear relationship model in which the number of *P. berghei *salivary gland sporozoite per individual *An. stephensi *mosquito linearly related to the number of oocysts per mosquito [40]. Although it describes better at low oocyst numbers (less than 50 per mosquito), according to this model, 45% reduction in oocyst number corresponds to 45% reduction in salivary gland sporozoites, matching well with the pattern observed in the present study. Therefore, the observed change in the reduction rate of parasite number may not be relevant to prolonged impairment of apicoplast replication, but a consequence of the density-dependent transition during late life-stage in the mosquito.

It has been reported that AZM has schizontocidal activity against *P. falciparum *[20], *Plasmodium vivax *[23] and *P. berghei *[19], implying that AZM may reduce transmission, to some extent, by killing progenitor cells of gametocytes. Although available reports on gametocytocidal effect of AZM against human malaria are limited, AZM has been reported to have a small impact on reducing *P. vivax *gametocytes [41], but almost no impact on *P. falciparum *gametocytaemia [42]. The latter case is consistent with the observation that *P. falciparum *gametocytes are resistant to most schizontocidal drugs [27]. In terms of gametocyte reducing activity, the impact of AZM on *P. berghei *gametocytaemia would be similar to that on *P. falciparum*, rather than *P. vivax*. In the present study, we focused on the activity of AZM against mosquito stages of *P. berghei*, and demonstrated that AZM reduces sporozoite burden in salivary glands, independently of its gametocytocidal effect. Coleman *et al *reported that several compounds which had shown transmission-blocking activity against *P. berghei *were also effective against both *P. falciparum *and *P. vivax *[5,12], suggesting that *P. berghei *model would be useful for screening of candidate compounds for a transmission-blocking drug for human malaria. Taken together, AZM would be promising for suppressing mosquito stage development of *P. falciparum *and *P. vivax*, and would deserve further study.

In the present study, AZM treatment resulted in 79% and 50% reduction in sporozoites in the midgut and salivary glands, respectively, whereas prevalence of mosquitoes was not affected. Considering that inocula of only 10 sporozoites in wide range of *Plasmodium *spp. can be infectious to their vertebral hosts, the prevalence rather than the infection intensity of mosquitoes would be a suitable parameter for evaluating any transmission-blocking strategy [40]. However, it has been proposed by Medley *et al *[43] that the impact of transmission-blocking substances appears mainly on reduction in infection intensity, but not on that of prevalence under conditions of high oocyst intensities, whereas a rapid reduction in prevalence occurs under conditions of low oocyst intensities. The authors claim that their data derived from several *Plasmodium *spp. including *P. falciparum *and *P. vivax*, may afford broad applicability. Their observations have been supported by Sinden *et al *[40], who reported a saturating relationship between mean oocyst numbers and mean salivary gland sporozoite numbers, implying that the impact of 50% reduction in oocyst number becomes evident in reduction of salivary gland sporozoite when oocyst burden is low (less than 50 oocysts per mosquito). Furthermore, the authors also demonstrated that a 90% blockade in oocyst numbers gives no effect on prevalence of infected mosquitoes when mean oocyst numbers are high (more than 100), whereas significant reduction in prevalence is expected when oocyst load is very low (less than 10). Taking these information into account, the inhibitory effect of AZM on *P. berghei *development in the mosquito observed under conditions of high parasite load may allow to speculate that AZM would reduce both infection intensity and prevalence of the mosquitoes that fed blood from individuals carrying gametocytes of human malaria parasites under field conditions where parasite loads of *P. falciparum *or *P. vivax *are significantly lower than those in this study [44-46]. In order to examine that possibility, further studies on transmission blocking effect of AZM on *P. berghei *under conditions of low parasite loads are underway.

The dose of AZM administered to mice in this study (400 mg/kg) was much higher than those used for human clinical practice (10-40 mg/kg) [17,19,35,47]. It has been reported that there are significant interspecies difference in pharmacokinetic parameters of AZM between mice and human [48-50]. The dose and peak concentration of AZM required for efficacy in murine models are considerably greater than those in human due to the higher rate of AZM clearance in mice (*e.g*. much shorter half-life and greater clearance rate in mice than in human) [48-50]. Therefore, the results obtained by the murine model in this study do not suggest that administration of 400 mg/kg of AZM would be necessary for suppression of the mosquito stage development of human malaria parasites.

The precise mechanism of the effect of AZM on apicoplast replication has not been fully elucidated. Sidhu *et al *[15] reported an intensive study using selected AZM-resistant *P. falciparum *and proposed that the anti-malarial properties of AZM are a result of its binding to the apicoplast 50 S ribosomal subunit and inhibiting protein synthesis in this organelle. Therefore, it will be necessary to investigate the inhibitory effect of AZM on the mosquito-stage development in genetically modified *P. berghei *that have various mutations in the genes coding for apicoplast ribosomal proteins. If binding of AZM to the apicoplast ribosomal protein leads to inhibition of both apicoplast replication and sporogony in the mosquito, it could be confirmed that the apicoplast is the target of AZM.

Inhibitory mechanism of AZM on gametocyte-ookinete transformation could not be explained by the suppression of apicoplast replication, because apicoplast does not replicate at this phase. A possible explanation would be that AZM may block the fatty acid synthesis pathway within the apicoplast, causing the parasite to run out of material necessary for the drastic membrane biogenesis and reorganisation to form machinery for motility and invasion, during gametocyte-ookinete transformation [33].

## Conclusions

It is a new finding that AZM reduces *Plasmodium *sporozoite production in the mosquito, the mechanism of which would not depend on gametocytocidal effect, but be caused by suppression of apicoplast replication and an uncharacterised effect on gametocyte-ookinete transformation. In conclusion, clear advantages of AZM include; 1) a unique feature of multi-targeting at blood stage (treatment), liver stage (prophylaxis), and mosquito stage (transmission blocking), possibly by a common mechanism of action including the inhibition of apicoplast replication, and 2) an excellent safety profile that allows AZM to be given to children and pregnant women. Both of these advantages would warrant further investigations of AZM, especially on its prevalence-reducing efficacy under conditions of low parasite loads. Thereafter, AZM would be tested for the substantial transmission-blocking activity against human malaria parasites, in the expectation of a new drug which would be effective in reducing malaria morbidity and mortality and inhibit the spread of drug-resistant parasites in malaria endemic areas.

## Competing interests

The authors declare that they have no competing interests.

## Authors' contributions

SS and MA conceived and designed the experiments. SS and YO performed the experiments. SS, YO, TK, and YT analysed the data. SS and MA wrote the paper. All authors read and approved the final manuscript.

## Supplementary Material

Additional file 1 Primer sets for *Plasmodium berghei *organelle-specific genes Click here for file
