# Supplementary material for: Suppressive effect of azithromycin on Plasmodium berghei mosquito stage development and apicoplast replication
Source: Malar J. 2010 Mar 10;9:73. doi: 10.1186/1475-2875-9-73 (PMC2846956; doi:10.1186/1475-2875-9-73)
Supplement: Additional file 1 — Primer sets for Plasmodium berghei organelle-specific genes [file 1475-2875-9-73-S1.DOC]

Additional file 1: Primer sets for *Plasmodium berghei* organelle-specific genes

| Organelle | Gene | Accession No. | Forward primer (5’-3’)  Reverse primer (5’-3’) | Product size |
| --- | --- | --- | --- | --- |
| Apicoplast | *tufA* | DQ414662 | TGTGCACATATAGATTGTCCTGG  TAGCTATATCCATTTGTGTAGCACC | 79 |
| Mitochondrion | *cytb* | DQ414645 | TGGGGACAAATGAGTTACTGG  CAGTGTATCCTCCACATAACCAA | 94 |
| Nucleus | *fabI* | AY423069 | GGATCTGTTGCTTCATTTCTTTTATCA  AATCCGTTATCCACATATATTGTTTGAC | 77 |
